# Adapting the TeamSTEPPS team performance observation tool for dyadic interprofessional VR simulations (vTPOT): a multi-step validation study

**DOI:** 10.1186/s41077-026-00431-0

**Published:** 2026-03-12

**Authors:** Marie Lehmann, Stefanie Hinz, Alexander Zamzow, Jan Mikulasch, Horst Poimann, Joy Backhaus, Marvin Mergen, Tobias Mühling

**Affiliations:** 1 Institute of Medical Teaching and Medical Education Research, University Hospital Würzburg, Josef-Schneider-Str. 2, Würzburg, 97080 Germany; 2 Department of Anesthesiology, Intensive Care Medicine and Emergency Medicine, Caritas Hospital Saarbrücken, Saarbrücken, Germany; 3 Department of Internal Medicine I, Intensive Care Unit, University Hospital Würzburg, Würzburg, Germany; 4 TeamSTEPPS Committee for German-Speaking Countries, Würzburg, Germany; 5 Department of Anesthesiology and Intensive Care Medicine, Saarbrücken Medical Center, Saarbrücken, Germany; 6 Department of Pediatric Oncology and Hematology, Faculty of Medicine, Saarland University, Homburg, Germany

**Keywords:** Interprofessional teamwork, TeamSTEPPS, Team Performance Observation Tool, TPOT, vTPOT, virtual reality, validity evidence, argument-based validation, emergency medicine education, medical simulation

## Abstract

**Background:**

Interprofessional teamwork is a key determinant of patient safety in medical emergencies, yet its structured assessment remains resource-intensive and is rarely integrated into undergraduate curricula. Immersive virtual reality (VR) offers scalable environments for training and assessing teamwork but introduces contextual constraints such as dyadic team structures, avatar-mediated interaction, and limited non-verbal cues. Established assessment tools, including the Team Performance Observation Tool (TPOT), have not been systematically adapted or validated for these VR-specific conditions.

**Methods:**

We adapted the TPOT for dyadic VR-based emergency simulations (vTPOT) through expert-driven item revision, development of behavioral anchors, and a two-round modified Delphi process with 15 interprofessional experts. No new VR-specific items were introduced, instead, items that could not be meaningfully observed in immersive VR were removed or consolidated to preserve conceptual stability. Five trained raters independently evaluated 21 VR simulation videos from interprofessional dyads (medical and nursing students) using both the original TPOT and the vTPOT. Following Kane’s argument-based validation framework, we examined evidence for scoring (content validity, association with the original TPOT), generalization (internal consistency, interrater reliability), extrapolation (associations with teamwork perceptions and objective medical performance), and implications (written qualitative responses from raters) on potential unintended effects and implications for feedback and assessment).

**Results:**

The final vTPOT comprises 15 behaviorally anchored items across five teamwork domains. Internal consistency was high (Cronbach’s α = 0.92), and interrater reliability was moderate to substantial (intraclass correlation coefficient = 0.68). vTPOT scores showed strong convergence with the original TPOT (*r* = 0.92) and with participants’ teamwork perceptions (T-TPQ; ρ = 0.64 and 0.80 for nursing and medical students, respectively). Higher vTPOT scores were positively associated with objectively measured medical performance in VR scenarios (*r* = 0.54). Raters reported that the vTPOT supported structured observation and feedback, while also highlighting context-specific limitations and potential unintended effects.

**Conclusions:**

The vTPOT appears to be a feasible and reliable instrument for assessing interprofessional teamwork in dyadic VR simulations within the studied context. Initial validity evidence supports its intended use for low-stakes assessment and research. Further studies are needed to examine construct structure and extend validation to additional contexts, populations, and outcomes to strengthen the overall validity argument.

**Supplementary Information:**

The online version contains supplementary material available at 10.1186/s41077-026-00431-0.

## Background

 Interprofessional teamwork is critical for patient outcomes, particularly in emergency medical contexts [1, 2]. Despite its importance, structured interprofessional team training is scarce in undergraduate education and clinical practice [3, 4], largely due to the substantial personnel and resource demands of effective training [5, 6]. Virtual reality (VR)–based environments are emerging as scalable, resource-efficient formats that can facilitate training [7, 8] and assessment [9, 10] of emergency medical competencies. While validated tools exist for objective assessment of teamwork quality in conventional simulation [11, 12], instruments tailored to VR are largely lacking. Importantly, unlike clinical knowledge or procedural skills, teamwork in VR poses unique challenges: Team-capable VR emergency medical environments vary regarding interfaces (e.g., menu-driven vs. speech-based [13]), haptics (solely via VR controllers [14, 15] or additional haptic devices [16]), limited non-verbal communication with team members, and restricted rater observation due to head-mounted displays [17]. Consequently, most studies so far have relied on perceived teamwork measures [17], highlighting the need for objective VR-adapted assessment tools.

The TeamSTEPPS framework [18] provides a foundation for teamwork education and assessment, with the Team Performance Observation Tool (TPOT) as objective assessment tool, which is particularly comprehensive compared to other tools [19]. In analog simulations, TPOT can achieve high internal consistency and substantial interrater reliability especially when supplemented with behavioral anchors [19–21]. Scores correlate with clinically meaningful outcomes, such as reduced team errors and faster performance, and improve following structured TeamSTEPPS training [20, 22–24]. Despite strong evidence supporting TPOT in conventional simulations, modifications to account for dyadic, avatar-mediated VR interactions with the above-mentioned constraints are necessary. However, to date only one study has adapted TPOT for VR- or computer-based simulations. The resulting version of the TPOT demonstrated strong content validity and interrater reliability within its VR scenarios, but its highly scenario-specific behavioral anchors strongly limit generalizability [25].

To address the unique characteristics of immersive VR teamwork, we adapted the TPOT by revising item content and structure and by developing corresponding behavioral anchors to enhance clarity, observability, and interrater reliability. The result is a scenario- and platform-agnostic tool designed to assess dyadic interprofessional teamwork in VR (vTPOT). Following Kane’s argument-based validation framework [26, 27], valid score interpretation requires evidence supporting each inference in the chain from observation to decision making. Because immersive VR substantially alters the context in which teamwork behaviors occur, none of the original TPOT’s validity assumptions can be presumed to transfer to dyadic, avatar-based VR settings.

In line with Kane’s argument-based validation framework, we explicitly define the intended interpretation and use of vTPOT scores: The vTPOT is conceptually intended to support summative evaluation of teamwork performance in dyadic VR simulations. However, the present study was deliberately designed to generate initial validity evidence for low-stakes and research applications, such as evaluating teamwork training effects, supporting formative feedback and informing the pedagogical design of VR-based scenarios. Accordingly, we conducted a structured, multi-step validation process to examine whether the adapted instrument supports these intended interpretations. This included evidence related to (1) scoring (clarity, relevance, and observability of items and anchors), (2) generalization (stability and consistency of ratings across raters and scenarios), and (3) extrapolation (the extent to which vTPOT scores reflect core teamwork competencies in immersive VR settings). We also explored aspects of (4) implications, mainly at the level of rater use and perceived fairness within the training context, acknowledging that broader impacts on clinical performance or patient outcomes lie beyond the scope of the present study.

## Methods

### Study context

The present adaptation of the TPOT for VR scenarios involving dyadic teams, along with its subsequent validation, was part of a study on the training and assessment of interprofessional teamwork (VISTA study) that was conducted between May 2024 and October 2025 at four medical faculties in Germany (Hannover Medical School, Saarland University Faculty of Medicine, Medical University of Münster, and University Hospital Würzburg). The detailed study procedure is described in the research protocol [28]. In summary, teams consisting of one nursing and one medical student completed three different interprofessional VR-based scenarios (each lasting 30 min) on three separate dates (day 1, 8 and 15): (1) esophageal variceal bleeding due to ethyl-toxic liver cirrhosis, (2) exacerbated chronic obstructive pulmonary disease, and (3) tachycardic atrial fibrillation due to complicated urinary tract infection. The contents of these scenarios and the indicated medical actions had been described in detail in previous studies [14, 29]. While no structured input on teamwork was provided on day 1 (corresponding to a pre-test), participants watched a 30-minute training video based on the TeamSTEPPS guidelines [18], adapted for the VR context on day 8 (corresponding to a post-test). It included practical recommendations and video examples illustrating key team interactions such as check-backs, huddles, feedback, and the Two-Challenge Rule. Participants were able to communicate via voice-over internet protocol (IP) and interacted with each other through avatars during the scenarios (e.g., handing over equipment or demonstrating findings on a screen). On day 15, similar to day 1, no structured input was provided, allowing assessment of how well the content delivered on day 8 was retained. For gathering evidence supporting interpretations of teamwork scores as well as blinded and objective assessment of teamwork, first-person video recordings including audio tracks were captured from both participants. Collected demographic variables comprised age, gender, and self-reported prior experience with non-immersive desktop-based 3D applications (such as 3D video games) and virtual reality (VR).

### Content adaptation and content validity via modified delphi process (Scoring)

**Fig. 1:**
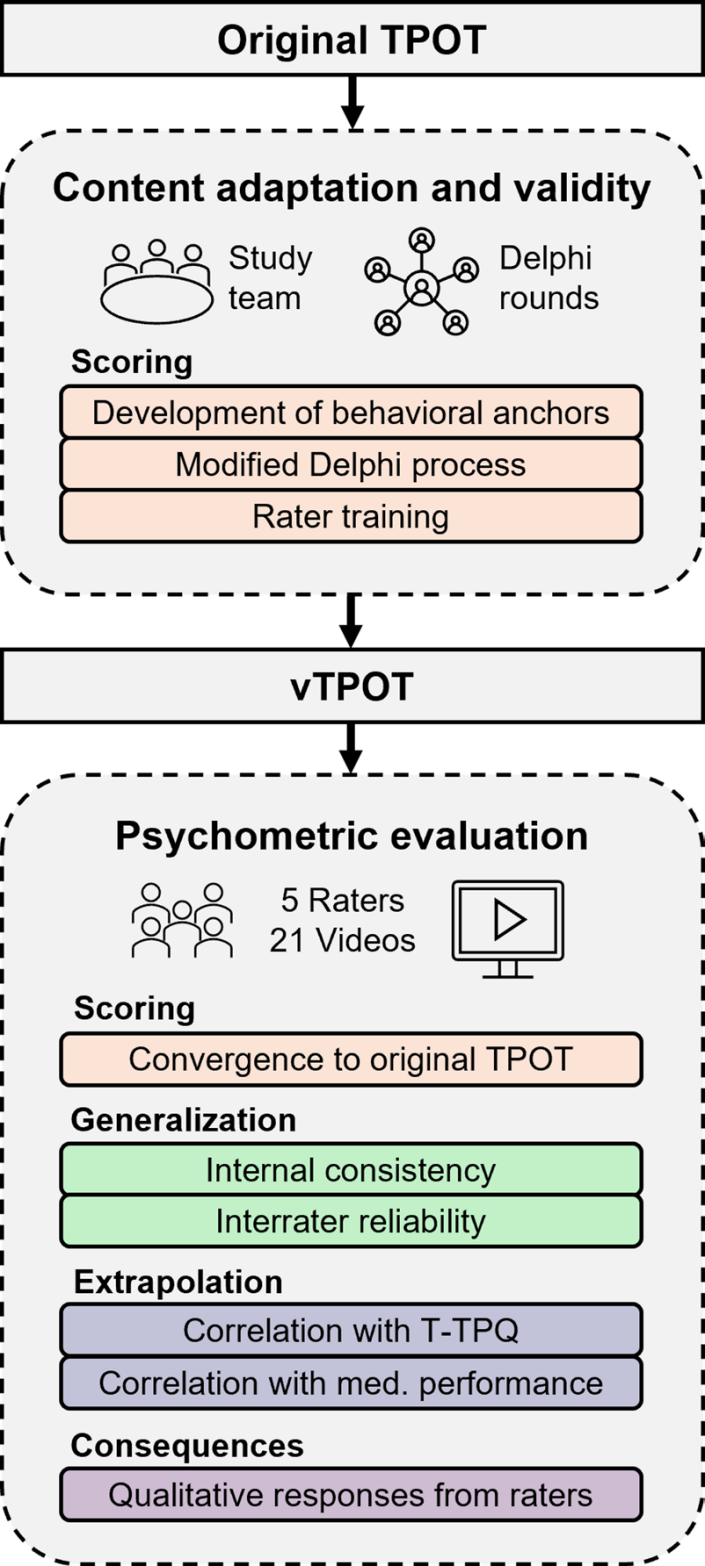
Development and initial validation of the vTPOT. Flow-chart depicting the adaptation of the original TPOT for use in immersive VR simulations and its initial validation. The upper section shows content adaptation and scoring validity through study team review, Delphi rounds, behavioral anchors, and rater training. The lower section summarizes psychometric evaluation, including scoring convergence with the original TPOT, generalization (internal consistency, interrater reliability), extrapolation (correlations with T-TPQ and medical performance), and consequences (written qualitative responses from raters). Colors correspond to Kane’s validity inferences: scoring (red), generalization (green), extrapolation (blue), consequences (purple)

To support the scoring inference, the TPOT was adapted for assessing teamwork in immersive, dyadic VR scenarios through a structured, multi-step content adaptation process (Figure 1) . Guided by the TeamSTEPPS 3.0 framework, all 23 original TPOT items were reviewed and preliminarily modified to account for VR-specific contextual constraints, including reduced nonverbal communication, avatar-mediated interaction, and two-person team structures. For each modified item, behavioral anchors (scores 5, 3, 1) were developed to enhance scoring objectivity. Rather than introducing items tailored to specific VR functionalities—which evolve rapidly and risk becoming obsolete—we focused on removing or consolidating TeamSTEPPS elements that cannot currently be meaningfully operationalized or observed in immersive VR or dyadic teams. This approach was chosen to preserve conceptual stability and support applicability across platforms and future technological developments.

Initial adaptations were drafted independently by four experts—a neurosurgeon and certified TeamSTEPPS trainer (HP), a specialist in internal medicine and medical education (TM), an intensive care nurse with additional training in medical education (JM), and the study coordinator (ML, skills-lab tutor with 2 years experience). Adaptations were consolidated through a structured consensus workshop.

This version underwent a two-round modified Delphi process with 15 interprofessional experts. Eligibility criteria required panelists to be licensed physicians or nurses with at least one of the following: (1) formal medical education training, (2) certification in emergency medicine (physicians) or emergency nursing (nurses), or (3) ≥ 50 h of experience facilitating simulation-based team training. Each item was presented together with its proposed wording, behavioral anchors, and VR example videos to sensitize panelists to context-specific observability constraints. In both rounds, panelists rated relevance, clarity, and contextual appropriateness on a 4-point Likert scale and provided qualitative comments for revision.

Consensus was defined a priori as ≥ 75% agreement (rating ≥ 3) and IQR ≤ 1. Items not meeting criteria were revised and re-evaluated in round 2. A final consolidation meeting with two additional TeamSTEPPS trainers (RF, CK, see acknowledgement) who were not part of the Delphi panel ensured conceptual integrity and alignment with the TeamSTEPPS framework. This process resulted in a VR-adapted, behaviorally anchored version of TPOT (vTPOT).

### Rater training, response process, and rating procedure (Scoring)

To further support the scoring inference by reducing rater variance, five expert raters participated in a standardized two-day training delivered by certified TeamSTEPPS instructors (RF, CK). Raters included one board-certified internist with expertise in medical education (TM), one clinician with training in emergency medicine and expertise in medical education (MM), one clinician with training in emergency medicine and prior professional experience as a registered nurse (SH), one clinician with specialization in medical education (AZ), and one experienced skills-lab tutor (ML). Training consisted of annotated VR example videos, calibration discussions and consensus-building exercises focused on ambiguous behaviors in dyadic VR interaction. During training, the raters developed and agreed upon a supplemental rater codebook, specifying additional consensus rules for items identified as context-sensitive or ambiguous. This codebook supported rating consistency and is provided in Supplementary Table 1. For selected items (3.2 and 3.3), intermediate qualitative descriptors were included in the rater codebook to support calibration in ambiguous cases. The numerical rating scale remained unchanged (5, 3, 1), and no additional scoring categories were introduced.

### Sampling strategy, correlation with the original TPOT and reliability assessment (Scoring and Generalization)

To support the generalization inference, 21 VR recordings were selected via a blueprint-guided sampling strategy from the VISTA study to ensure variation in clinical content and teamwork behaviors across three standardized internal medicine emergency scenarios. All five raters independently evaluated each video blinded to participants and study time point (d1, d8, or d15) using both the vTPOT and the original TPOT in alternating order to mitigate sequence effects. For the correlation with the original TPOT, individual items in both the original TPOT and the vTPOT were aggregated into facet-level scores (unweighted mean of item scores within each facet), and Pearson correlations were then calculated across all videos, raters and facets to assess convergence between the two instruments. Regarding interrater reliability, after 12 videos (partial round 1), an interim analysis was conducted by calculating intraclass correlation coefficients (ICCs). Based on identified inconsistencies, selective refinements were made to the codebook and verified through calibration on an additional video, which was one of the 9 videos in partial round 2. Subsequently, the remaining 8 videos of partial round 2 were rated, and ICCs for all 9 videos were recalculated to evaluate reliability stability across rounds. Internal consistency of the vTPOT was assessed via Cronbach’s α.

### Responsiveness as well as associations with subjective teamwork quality and medical performance in VR scenarios (Extrapolation)

Responsiveness was assessed across the three VISTA sessions (days 1, 8, and 15), with a brief teamwork training delivered immediately before the day‑8 scenario, which was expected to improve team performance. Convergent validity was evaluated by correlating vTPOT scores with teams’ self-reported teamwork quality, using the teamwork perceptions questionnaire (T‑TPQ) based on the TeamSTEPPS framework. Criterion-related validity was assessed via correlations between vTPOT scores and objective medical performance in the VR scenarios, automatically captured by the VR software [9]. The program recorded all relevant user actions and scenario-specific target parameters (e.g. mean arterial pressure > 65mmHg) according to scenario-specific checklists established by the authors based on professional society guidelines and previously verified against manual scoring [9]. Based on this, an overall medical performance score between 0 and 100% was calculated for each scenario completed by a team.

### Consequential validity by qualitative rater feedback (Implications)

To explore interpretability, perceived utility and fairness of the vTPOT within formative and summative educational settings, raters were asked to respond to written, structured interview questions. Both the questions and the responses were exchanged in writing, rather than conducted orally, ensuring a standardized and documented format for all raters. The questions addressed three themes: (1) intended consequences, (2) unintended or problematic effects, and (3) consequences for feedback and assessment. Written responses were then thematically analyzed by the first author (ML) [30]. This approach provided early insights into the potential educational value and acceptability of the vTPOT, as well as areas for further refinement.

### Software and hardware

All videos used for the validation analyses were recorded from the participants’ desktop view during VR sessions using the open-source software OBS Studio (Version 30.0.1; OBS Project, https://obsproject.com). The VR scenarios were implemented in STEP-VR (Version 1.0; ThreeDee GmbH, Munich, Germany, since 10/2025: OrangeWhip Interactive, Munich, Germany), a multiuser simulation platform jointly developed by a 3D visualization company and the University Hospital of Würzburg. The software enabled participants to select medical or nursing avatars with stylized facial features and to interact synchronously within shared emergency scenarios.

The VR hardware setup at the primary study site consisted of two OMEN by HP 17-ck0075ng laptops (Intel Core i7-11800 H, NVIDIA GeForce RTX 3070 Laptop GPU, 8 GB GDDR6) connected to two Meta Quest 3 head-mounted displays. This configuration ensured stable operation of STEP-VR at high graphical settings with frame rates consistently above 60 fps. At the additional study sites, Meta Quest 3 head-mounted displays and comparable laptop configurations were used to ensure equivalent system performance and user experience across locations.

### Statistical analyses

Descriptive statistics were calculated and reported as mean ± SD. Data distributions were assessed using the Shapiro–Wilk test. For normally distributed variables (TPOT and vTPOT scores, medical performance), group differences were analyzed using independent-samples t-tests or one-way ANOVA, as appropriate. For significant ANOVA results, post hoc pairwise comparisons were performed using t-tests with FDR correction according to Benjamini–Hochberg. Associations were analyzed using Pearson’s correlations (r). For non-normally distributed variables (T-TPQ scores), Wilcoxon rank-sum tests and Spearman rank correlations (ρ) were applied. These analyses and visualizations were performed in GraphPad Prism (Version 10.1.2).

Little’s Missing Completely At Random (MCAR) test was used to evaluate whether missing values occurred completely at random. Interrater reliability was examined using intraclass correlation coefficients (ICC) based on a two-way random-effects model for agreement. ICC analyses were conducted with the R packages Coefficients of Interrater Reliability – Generalized for Randomly Incomplete Datasets (v0.2.3) and, where required, Various Coefficients of Interrater Reliability and Agreement (v0.84.1). ICC(2) values were interpreted according to established guidelines (< 0.40 poor, 0.40–0.75 fair to good, > 0.75 excellent) [31, 32]. Internal consistency was assessed using Cronbach’s alpha (α), with α ≥ 0.70 considered acceptable.

## Results

### Content adaptation and content validity via modified delphi process and convergent validity with the original TPOT (Scoring).

During adaptation by the author team, 9 of the 23 items in the original TPOT (1a, 1d, 3a, 3b, 3c, 4b, 4c, 4e, 5d) were excluded due to limited relevance in the VR-based dyadic team context. An additional 6 items (1b, 2b, 3d, 3f, 5b, 5c) were substantially revised to reflect VR-specific constraints and dyadic interactions. Behavioral anchors were developed for the resulting 14 items. In Delphi round 1, consensus (≥ 75% agreement and IQR ≤ 1) was reached for 11/14 items and 12/14 behavioral anchors. Items and anchors not reaching consensus were revised. The removal of one of the original items (4b) also failed to reach consensus, and the item was therefore reintroduced into the item set. In round 2, 14/15 items and 14/15 anchors achieved consensus. The remaining item (4b) was further refined and reviewed by all experts. Final expert discussion resulted in minor rewording of six items to ensure clarity and alignment with the TeamSTEPPS framework. The final vTPOT version included 15 items across the domains Team Structure (2), Communication (4), Leadership (3), Situation Monitoring (3), and Mutual Support (3) (Table 1). The derivation from the original TPOT items and the rationale for removed or consolidated items are presented in Supplementary Table 4.

**Table 1:** Final item set of the vTPOT. The table displays the 15 items of the Virtual Team Performance Observation Tool (vTPOT), organized by TeamSTEPPS domains and including behavioral anchors (5/3/1). Items represent observable teamwork behaviors adapted for dyadic, VR emergency scenarios following a modified Delphi process

1. **TEAM STRUCTURE**
1.2 Maintains regular contact with the other team member, taking the role responsibilities into account.
5 = Actively keeps track of the other team member, initiates interactions, and considers role responsibilities
3 = Limited active contact; responds to contact attempts; partially considers role responsibilities
1 = No active contact; communication limited to essentials; misunderstanding of roles
1.1 Introduces with name and role, and asks for the name and role of the other team member if not spontaneously provided.
5 = Both team members introduce themselves with name and role
3 = Introduction is one-sided or important information is missing
1 = No introduction occurs
2. **COMMUNICATION**
2.1 Provides brief, clear, specific, and timely information to team members.
5 = Information is shared clearly, concisely, and promptly
3 = Information is shared delayed, verbose, or unnecessarily complex
1 = Information is not shared
2.2 Seeks information from all relevant sources.
5 = Relevant sources are adequately considered in a clinically appropriate manner
3 = Relevant sources are partially considered or only the most obvious ones are used
1 = Even obvious sources are ignored
2.3 Uses check-backs to verify information that is communicated.
5 = Check-backs are consistently used for error-prone information (e.g., dosages)
3 = Check-backs are used occasionally or information is not fully confirmed (no complete closed-loop)
1 = Check-backs are not used
2.4 Uses structured handover techniques when transferring patient information.
5 = Structured handover (e.g., SBAR) is fully and correctly applied
3 = Structured elements are inconsistently or incompletely applied
1 = No structured handover is used or handover does not occur despite being required
3. **LEADERSHIP**
3.1 Delegates tasks or assignments, as appropriate.
5 = Tasks aligned with the team member’s role are consistently delegated
3 = Tasks are irregularly delegated or misaligned with the role
1 = No tasks are delegated
3.2 Conducts briefings, huddles, and debriefs.
5 = All tools are fully and appropriately used
3 = Tools are partially or inappropriately used (e.g., team members not included in huddle)
1 = Tools are not used
3.3 Models cooperative and attentive team behavior.
5 = Spoken points are actively acknowledged and constructively considered
3 = Statements are acknowledged but not addressed further
1 = Does not respond or responds unconstructively
4. **SITUATION MONITORING**
4.1 Regularly monitors the status of the patient.
5 = Actively checks patient status, e.g. vital signs and patient complaints
3 = Patient condition is addressed at least initially and once during scenario
1 = Patient condition is widely ignored
4.2 Observes team members to ensure safety and prevent errors.
5 = Team members are monitored for relevant actions and these actions are verbalized back
3 = Team members are partially observed and actions verbalized
1 = Team members are not monitored
4.3 Discusses treatment progress based on the patient’s status.
5 = Treatment goals are clearly defined based on patient status (e.g., using ABCDE) with necessary actions
3 = Patient status guides a rough direction for treatment
1 = No treatment goals are defined
5. **MUTUAL SUPPORT**
5.1 Provides task-related support and assistance when needed.
5 = Support is consistently offered in appropriate situations and matches the team member’s competence
3 = Support is occasionally or insufficiently offered
1 = No support is offered
5.2 Provides timely and constructive feedback to team members.
5 = Feedback consistently meets good-feedback criteria (timely, constructive, action-oriented)
3 = Feedback only partially meets good-feedback criteria
1 = Feedback is absent or inappropriate, even when warranted
5.3 Alerts to patient deterioration and raises concerns a second time if ignored (Two-Challenge Rule).
5 = Two-Challenge Rule is applied appropriately
3 = Concerns are vague or only raised once despite lack of acknowledgement
1 = Concerns are not raised despite the situation would require it

Correlation between vTPOT and the original TPOT scores was high (*r* = 0.92, *p* < 0.001), suggesting that the fundamental domains of teamwork were preserved.

### Reliability assessment (Generalization)

During psychometric evaluation, 21 videos from interprofessional dyads were rated, comprising data from 42 participants (21 nursing and 21 medical students). Nursing students were slightly older on average than medical students (25.8 ± 10.3 vs. 24.0 ± 1.8 years) and predominantly female (95% vs. 71%). Across both groups, prior exposure to 3D applications was limited, with the majority reporting no or infrequent use. Similarly, most participants reported little cumulative VR experience before the study, with over half in each group indicating no prior VR exposure (Table 2).

**Table 2:** Participant characteristics. Demographic characteristics and prior experience with 3D desktop and virtual reality applications of nursing and medical students. Data are presented as mean ± SD or n (%). VR: virtual reality

Characteristic	Nursing students (*n* = 21)	Medical students (*n* = 21)
Age, years	25.8 ± 10.3	24.0 ± 1.8
Gender, n (%)		
Female	20 (95)	15 (71)
Male	1 (5)	6 (29)
Diverse	0 (0)	0 (0)
Frequency of 3D application use, n (%)		
Never	12 (57)	15 (71)
< 1x / month	0 (0)	3 (14)
1x / month to 1x / week	1 (5)	0 (0)
Several times per week	3 (14)	0 (0)
Daily	0 (0)	0 (0)
Not reported	5 (24)	3 (14)
Cumulative VR experience prior to study, n (%)		
None	11 (52)	13 (62)
0–1 h	2 (10)	1 (5)
1–5 h	3 (14)	3 (14)
5–20 h	0 (0)	1 (5)
> 20 h	5 (24)	3 (14)

The adapted vTPOT demonstrated acceptable internal consistency across domains, with Cronbach’s α increasing from 0.90 in partial round 1 to 0.92 in partial round 2 after codebook refinements.

Interrater reliability (ICCs) improved from moderate agreement in partial round 1 (vTPOT: 0.54; 95%CI [0.43, 0.64]; original TPOT: 0.49 [0.38, 0.60]) to substantial agreement in partial round 2 (vTPOT: 0.68 [0.61, 0.75]; original TPOT: 0.64 [0.56, 0.71]), indicating increased stability and consistency across raters and scenarios. Domain-specific analyses revealed that the vTPOT demonstrated equal or higher interrater reliability than the original TPOT in all domains, with noticeable improvements after codebook refinement in partial round 2. Resulting reliability was strongest and most stable in Team Structure (0.88 [0.76,0.95]), moderate in Communication and Leadership (0.66 [0.51,0.81] and 0.69 [0.55,0.82]), and lower but improving in Situation Monitoring and Mutual Support (0.51 [0.35, 0.72] and 0.41 [0.15,0.70]) with items that were more difficult to operationalize. Fig. 1

### Responsiveness, associations with teamwork perceptions and medical performance in VR scenarios (Extrapolation)

**Fig. 2:**
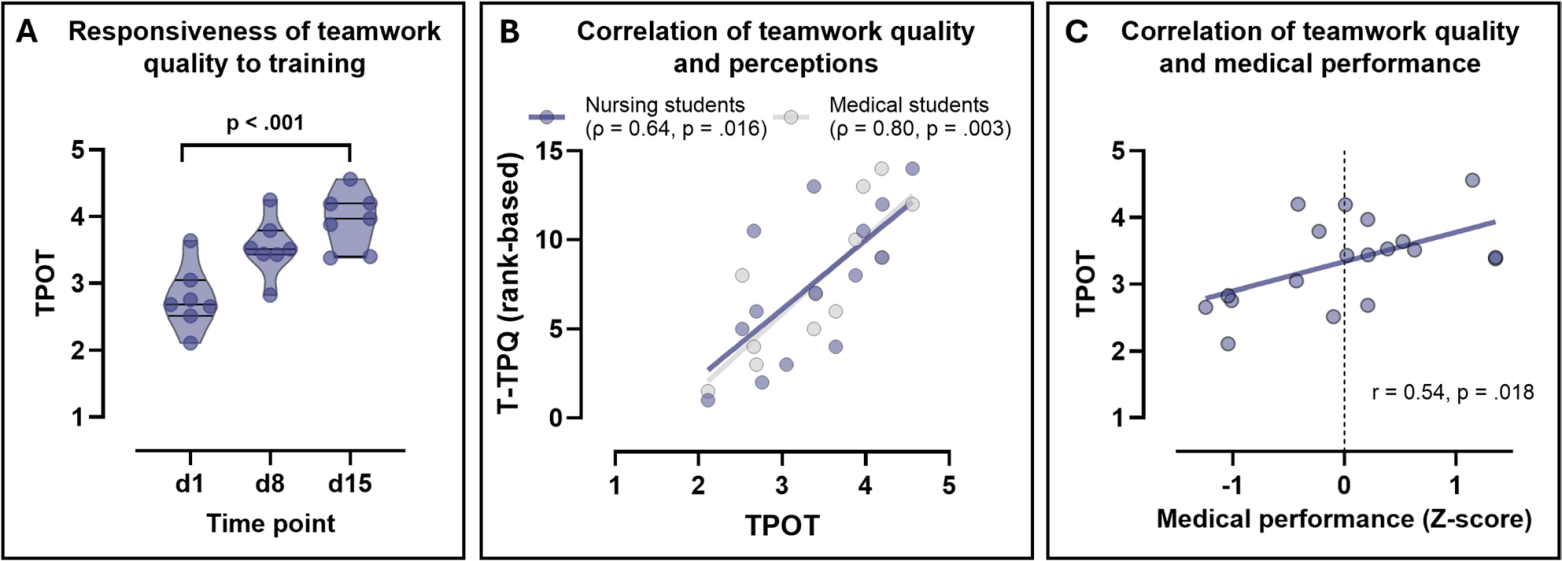
Responsiveness of the vTPOT to teamwork training and convergent and criterion-related validity evidence. **A**: Violin plots with individual data points, medians, and interquartile ranges showing improvements in vTPOT teamwork scores across study days (day 1, day 8 after brief teamwork training, and day 15). **B**: Scatterplots showing convergent validity between vTPOT scores and teamwork perceptions (T-TPQ). Because T-TPQ scores were non-normally distributed, rank-based correlations (Spearman’s ρ) are shown, and the T-TPQ axis represents ranked values, with separate regression lines for nursing students (blue) and medical students (gray). **C**: Scatterplot illustrating criterion-related validity, displaying the association between vTPOT scores and z-standardized medical performance across VR scenarios, with regression line

The vTPOT showed clear responsiveness to teamwork training. A one-way ANOVA indicated significant improvements in objectively rated teamwork quality from day 1 (mean 2.77 ± 0.47) to day 8 (mean 3.54 ± 0.42) and day 15 (mean 3.94 ± 0.43), all *p* < 0.001 (Fig. 2A). Post hoc t-tests with FDR correction confirmed significant increases from day 1 to day 8 and from day 1 to day 15, whereas the difference between day 8 and day 15 was not statistically significant.

Convergent validity evidence was observed through strong correlations between vTPOT scores and participants’ perceptions (T‑TPQ) with ρ = 0.64 (*p* = 0.016) for nursing students and ρ = 0.80 (*p* = 0.003) for medical students (Fig. 2B).

Criterion-related validity was indicated by a significant positive association between vTPOT scores and objectively measured medical performance in the VR scenarios (*r* = 0.54, *p* = 0.018), suggesting that higher teamwork quality was associated with higher-quality clinical task performance (Fig. 2C).

### Consequential validity by qualitative rater feedback (Implications)

Raters generally perceived the vTPOT as a helpful and structured tool for observing teamwork behaviors and supporting reflection. Intended consequences included improved recognition of key team dimensions, such as role-based delegation, communication, and mutual support, facilitated by the behavioral anchors and tool structure, which also supported specific feedback and self-reflection. Unintended or problematic effects involved occasional skipping of items due to scenario structure, challenges in rating asymmetric teams, and VR-specific limitations affecting perspective and communication. Some aspects of teamwork, such as shared team cohesion or enjoyment, were not fully captured, and a few raters reported minor concerns about subjectivity despite the anchors. Consequences for feedback and assessment were generally positive: raters expressed willingness to reuse the vTPOT for formative feedback, highlighting its clear structure and accessibility. Suggestions for improvement included conditional item presentation (e.g., rating handovers only if they occurred) and adaptations for cases of unequal team participation. Detailed results of the thematic analysis, including representative quotes and frequency counts, are provided in Supplementary Table 2.

## Discussion

This study adapted the established TPOT for use in immersive, dyadic VR simulations and generated initial validity evidence across Kane’s four inferences. Overall, the adapted vTPOT showed clear behavioral anchoring, moderate to substantial interrater reliability, high internal consistency, strong convergence with the original TPOT, and criterion-related alignment with medical performance and team member perceptions. Qualitative feedback further indicated that raters perceived the vTPOT as usable, fair, and supportive for rating and structured feedback in VR contexts.

To strengthen the scoring process, initial adaptations were conducted by a team of interprofessional experts and behavioral anchors were developed for each item to support consistent scoring [20]. Following established Delphi standards [33, 34], a two-round modified Delphi process with a diverse panel of 15 experts achieved consensus on 15 items and their anchors. Similar Delphi-based approaches were used in other teamwork tools, such as scales for obstetric emergencies [35] and the TeamUP rubric [36]. While the Team Emergency Assessment Measure (TEAM) has been adapted to VR with minor adjustments [37], this approach may be insufficient for the more behaviorally detailed TPOT, which is more comprehensive [19, 24], including explicitly teachable behaviors (e.g., huddles, Two-Challenge Rule, closed-loop communication) as well as items involving patients and their families. Consequently, during the adaptation process, several TPOT items were removed or consolidated to fit two-person VR scenarios—notably items on team assembly, patient/family inclusion, complex resource allocation/environment checks, and the DESC conflict script. While this streamlining focused the tool on directly observable behaviors, it may also have reduced content coverage, altered the construct being measured and thus impaired comparability with prior (analogous) studies. However, the strong convergent validity with the original TPOT indicated that the adapted tool captures core teamwork domains in VR in a manner consistent with the established tool. Future work could investigate measurement invariance through confirmatory factor analysis to determine, whether construct structure is preserved. Moreover, think-aloud protocols could further explore raters’ cognitive processes and decision-making during scoring.

Regarding the generalization of vTPOT scores, internal consistency of the vTPOT was high, aligning with prior TPOT applications in interprofessional simulations, which report similar α values of 0.92 [20] and 0.97 [21, 38] Our results demonstrated moderate to substantial interrater reliability (ICCs) across teamwork domains, with intraclass correlation coefficients ranging from 0.41 to 0.88 after codebook improvement. While the simulation-specific codebook facilitated higher interrater reliability in our dyadic VR scenarios, the need for such a codebook may vary depending on scenario complexity and rater experience. Two domains—Situation Monitoring and Mutual Support—showed comparatively lower agreement. Similar patterns have been reported in prior TPOT and TEAM research, where such constructs - often relying on subtle, non-verbal clues - exhibit lower sensitivity or reduced variability, particularly in high-performing teams or scenarios with limited opportunities to demonstrate such behaviors explicitly [24, 39]. Notably, ICCs from the adapted vTPOT were higher compared to the original TPOT version without behavioral anchors when the same VR recordings were rated. This aligns with other simulation-based studies where interrater reliability of TPOT ranged from 0.44 (measured as ICC) [19] and 0.46 [39] whereas values increased with use and specificity of behavioral anchors to 0.73 [20] and 0.85 [25] (measured as Cohen’s Κ). However, while such anchors may enhance reliability, a careful trade-off is necessary between anchor specificity and generalizability when adapting assessment tools to immersive VR settings. Future work should expand sampling to additional clinical contexts and larger professional groups and consider more sophisticated generalizability analyses to further strengthen evidence for score generalization [40].

With respect to extrapolation, high alignment with T‑TPQ scores indicated that observer-rated behaviors correspond well with teams’ own perceptions of their performance. While systematic comparisons between observer-based ratings and self-assessments of teamwork remain rare within the TeamSTEPPS framework, comparable correlations are reported from ad-hoc emergency teams using the TEAM checklist [41]. In another study, teamwork was rated higher by the team itself compared to faculty members with rating accuracy increasing with team performance [42]. It will be interesting to see whether such an association can be replicated in the final analysis of the VISTA study, when sample size is sufficient for a more detailed analysis. Criterion-related evidence indicated that higher vTPOT scores corresponded to better objectively measured clinical task performance within the VR scenarios, reflecting a relationship between observed teamwork behaviors and performance outcomes. While current approaches assessing teamwork in VR have not yet provided data on such outcome relationships [25, 37], similar associations have been observed between improved teamwork and clinical outcomes, such as reduced time to critical interventions [22] and reduced medical errors [20]. However, in another study assessing teamwork during neonatal resuscitation, no tool demonstrated a meaningful correlation with adherence to clinical guidelines, emphasizing the context-dependence of this relationship [19]. Ideally, future studies should extend validation to include actual workplace performance and, ultimately, measures of patient safety outcomes.

Our written qualitative responses from raters on implications indicate that the vTPOT is a practical and usable tool for structuring observation and reflection on teamwork, with clear behavioral anchors and suitability for both feedback and assessment purposes. Reported frequent skipping of items that are not relevant in specific scenarios may introduce some confusion for raters, although maintaining a sufficiently versatile item set is essential to ensure applicability across different use cases. Asymmetric team performance remains a known challenge for raters in conventional teamwork assessment tools as well [43], and the vTPOT is not unique in this regard. Because prior adaptations of teamwork assessment tools for VR contexts have not included qualitative evaluations from raters [25, 37], situating our findings within the existing literature is difficult and underscores the need for further inquiry in this area. A clear gap of our study is the missing qualitative assessment of team members, for example through interviews exploring their perception of the feedback that can be provided via the vTPOT [44] or an evaluation of resulting changes in their practice behavior (e.g. lower error rates) beyond the measured improvement in medical performance in the VR scenarios [45].

Taken together, the pattern of evidence suggests that vTPOT scores can be defensibly interpreted for low-stakes assessment and research applications in dyadic VR simulations. Scoring inferences are well supported by behaviorally anchored items and convergent validity, while generalization is reasonably substantiated through internal consistency and substantial interrater reliability. Evidence for extrapolation and implications inferences is promising but preliminary, relying on correlations with subjective teamwork perceptions and medical performance as well as qualitative rater feedback. These findings highlight both the strengths and boundaries of the current validity argument: while the tool captures core teamwork behaviors and is practically usable, broader sampling, diverse contexts, and longitudinal data are required to fully justify high-stakes use or to generalize beyond the studied VR scenarios. Table 3 summarizes the collected validity evidence, identifies remaining gaps, and outlines corresponding directions for future research.

**Table 3:** Validity evidence, remaining gaps and directions for future research. The table synthesizes collected validity evidence, remaining gaps, and priority directions for future research across the scoring, generalization, extrapolation, and implications inferences. Evidence is based on a multi-step validation process including a Delphi study, rater training, reliability analyses, correlations with teamwork perceptions and medical performance, and written qualitative responses from raters

Inference / Classical Validity Type	Tools & Results (Summary)	Potential Gaps in Validity Evidence	Examples for Future Research
Scoring (Content validity, Response process)	Two-round modified Delphi process with 15 interprofessional experts → final 15 items; ≥75% consensus; behavioral anchors developed. High convergence to original TPOT (*r* = 0.92). Anchors and training supported clear, consistent scoring.	Limited evidence on raters’ cognitive processes; no test–retest data; construct structure not formally examined.	Conduct think-aloud protocols to explore cognitive scoring processes; repeat ratings on identical videos to assess stability; perform confirmatory factor analysis to examine construct preservation.
Generalization (Interrater reliability, Internal consistency)	Interrater reliability ICCs improved from 0.54 → 0.68 after codebook refinements; internal consistency α = 0.90 → 0.92; five raters across three VR scenarios acquired from multiple institutions. Domain-specific ICCs strongest for Team Structure, moderate for Communication/Leadership, lower but improving for Situation Monitoring/Mutual Support.	Limited scenarios and raters; no longitudinal data; potential context-dependence of subtle behaviors.	Expand sampling to additional clinical contexts and professional groups; multi-institutional replication; longitudinal assessment; advanced generalizability analyses
Extrapolation (Convergent and criterion validity)	Strong correlations with T-TPQ perceptions: nursing students *ρ* = 0.64, medical students *ρ* = 0.80; criterion-related correlation with VR medical performance *r* = 0.54.	Small sample; no predictive validity; convergent validity limited to TPOT/T-TPQ; lack of real-world or patient-outcome data.	Link vTPOT scores to clinical placements, OSCE performance, or workplace outcomes; replicate in larger samples; correlate with patient safety or performance surrogates.
Implications (Consequential validity, Educational utility)	Written qualitative responses from raters indicated the tool is usable, fair, and supportive for structured observation and feedback; anchors improved clarity; scenario-specific adaptation suggestions noted.	Limited to rater perspective; no data on learners’ perceptions, educational impact, or unintended consequences; no evidence on behavior change or patient outcomes.	Conduct learner surveys/focus groups to capture perceptions and impact; assess effect of feedback on teamwork performance; monitor unintended effects in training and practice.

### Limitations

Several limitations pertain to the validation process itself. As validation is inherently iterative and context-dependent, the present study provides only an initial set of arguments across Kane’s inferences. Evidence for scoring, generalization, and extrapolation was necessarily bounded by the available data and by interpretive decisions during instrument adaptation, and several inferences remain only partially addressed. Importantly, implications evidence is limited to rater perspectives; although raters perceived the vTPOT as usable and fair, these insights provide only an incomplete view of potential intended and unintended effects. Learners’ perceptions of fairness, cognitive load, or training impact were not assessed and remain essential for a comprehensive evaluation of implications inferences.

Beyond these conceptual limits, several methodological and contextual factors should be considered. Ratings were based on first-person VR recordings, which inherently reduce peripheral and non-verbal cues and may restrict observability despite purposeful adaptation of the tool. All raters were highly familiar with TeamSTEPPS and the study context, which may have lowered variability and limits generalizability beyond similarly trained groups. Moreover, the validation was restricted to dyadic teams performing three internal-medicine emergency scenarios, which may not reflect performance in larger or more heterogeneous clinical teams and other professional subjects. Some retained items capture explicitly taught TeamSTEPPS behaviors (e.g., huddles, the Two-Challenge Rule), which may disadvantage learners trained under different teamwork frameworks. Finally, quantitative analyses relied on a limited number of video cases, and the qualitative component focused exclusively on raters, leaving open questions about learner-level consequences and downstream educational impact.

## Conclusions

Despite these limitations, our findings offer an initial but coherent validity argument, suggesting that the vTPOT may support informed decisions about VR teamwork performance and training effectiveness within similar dyadic VR settings. Nevertheless, broader sampling, verification of construct structure, and further examination of its relationship to team performance in clinical settings are required to strengthen the overall validity argument and to justify use in high-stakes contexts.

## Supplementary Information

Supplementary Material 1. Table S1: vTPOT Rater Codebook

Supplementary Material 2. Table S2: Qualitative Feedback of Raters.

Supplementary Material 3. Table S3: Aggregated Data on TPOT, Medical Performance and T-TPQ Scores.

Supplementary Material 4. Table S4: Comparison of TPOT and vTPOT Items.

## Data Availability

The underlying aggregated data for the 21 videos, including total TPOT rating scores from the five raters, medical performance scores, and T-TPQ scores stratified by profession, are provided in Supplementary Table 3. The original video recordings can be obtained from the authors upon reasonable request.
