# Supplementary material for: Adapting the TeamSTEPPS team performance observation tool for dyadic interprofessional VR simulations (vTPOT): a multi-step validation study
Source: Adv Simul (Lond). 2026 Mar 12;11:24. doi: 10.1186/s41077-026-00431-0 (PMC13023181; doi:10.1186/s41077-026-00431-0)
Supplement: Supplementary file 3 — Supplementary Material 3. Table S3: Aggregated Data on TPOT, Medical Performance and T-TPQ Scores. [file 41077_2026_431_MOESM3_ESM.docx]

| **Original TPOT** | **Adapted vTPOT** |
| --- | --- |
| **1. Team Structure** |  |
| **a. Assembles a team** | **Item 1a – removed**  **Comment:**  **This item was removed because team composition is predefined in scenarios involving two participants. Additional aspects of the original item are sufficiently captured within adapted Item 1.1.** |
| **b. Assigns or identifies team members’ roles and responsibilities** | **1.1 Introduces with name and role, and asks for the name and role of the other team member if not spontaneously provided.**  5 = Both team members introduce themselves with name and role  3 = Introduction is one-sided or important information is missing  1 = No introduction occurs |
| **c. Holds team members accountable** | **1.2 Maintains regular contact with the other team member, taking the role responsibilities into account.**  5 = Actively keeps track of the other team member, initiates interactions, and considers role responsibilities  3 = Limited active contact; responds to contact attempts; partially considers role responsibilities  1 = No active contact; communication limited to essentials; misunderstanding of roles |
| **d. Includes patients and families as part of the team** | **Item 1d – removed**  **Comment:**  **At present, VR simulations do not provide functionality (e.g., natural language processing) that would allow the observation of the behaviors targeted by this item in a meaningful and reliable manner.** |
| **2. Communication** |  |
| **a. Provides brief, clear, specific, and timely information to team members** | **2.1 Provides brief, clear, specific, and timely information to team members.**  5 = Information is shared clearly, concisely, and promptly  3 = Information is shared delayed, verbose, or unnecessarily complex  1 = Information is not shared |
| **b. Seeks information from all available sources** | **2.2 Seeks information from all relevant sources.**  5 = Relevant sources are adequately considered in a clinically appropriate manner  3 = Relevant sources are partially considered or only the most obvious ones are used  1 = Even obvious sources are ignored |
| **c. Uses check-backs to verify information that is communicated** | **2.3 Uses check-backs to verify information that is communicated.**  5 = Check-backs are consistently used for error-prone information (e.g., dosages)  3 = Check-backs are used occasionally or information is not fully confirmed (no complete closed-loop)  1 = Check-backs are not used |
| **d. Uses SBAR, call-outs, and handoff techniques to communicate effectively with team members** | **2.4 Uses structured handover techniques when transferring patient information.**  5 = Structured handover (e.g., SBAR) is fully and correctly applied  3 = Structured elements are inconsistently or incompletely applied  1 = No structured handover is used or handover does not occur despite being required |
| **3. Leadership** |  |
| **a. Identifies team goals and vision** | **Item 3a – removed**  **Comment:**  **This item is less relevant in two-person teams and is sufficiently covered by interim team discussions / huddles (adapted Item 3.2) and by verbalization of treatment progress (adapted Item 4.2).** |
| **b. Uses resources efficiently to maximize team performance** | **Item 3b – removed**  **Comment:**  **This item is less relevant in dyadic teams and is adequately represented through efficient diagnostic information gathering (adapted Item 2.2) and task delegation (adapted Item 3.1).** |
| **c. Balances workload within the team** | **Item 3c – removed**  **Comment:**  **This item is less relevant in two-person teams and is sufficiently addressed through task delegation behaviors captured in adapted Item 3.1.** |
| **d. Delegates tasks or assignments, as appropriate** | **3.1 Delegates tasks or assignments, as appropriate.**  5 = Tasks aligned with the team member’s role are consistently delegated  3 = Tasks are irregularly delegated or misaligned with the role  1 = No tasks are delegated |
| **e. Conducts briefs, huddles, and debriefs** | **3.2 Conducts briefings, huddles, and debriefs.**  5 = All tools are fully and appropriately used  3 = Tools are partially or inappropriately used (e.g., team members not included in huddle)  1 = Tools are not used |
| **f. Models teamwork behaviors** | **3.3 Models cooperative and attentive team behavior.**  5 = Spoken points are actively acknowledged and constructively considered  3 = Statements are acknowledged but not addressed further  1 = Does not respond or responds unconstructively |
| **4. Situation Monitoring** |  |
| **a. Monitors the status of the patient** | **4.1 Regularly monitors the status of the patient.**  5 = Actively checks patient status, e.g. vital signs and patient complaints.  3 = Patient condition is addressed at least initially and once during scenario  1 = Patient condition is widely ignored |
| **b. Monitors fellow team members to ensure safety and prevent errors** | **4.2 Observes team members to ensure safety and prevent errors.**  5 = Team members are monitored for relevant actions and these actions are verbalized back  3 = Team members are partially observed and actions verbalized  1 = Team members are not monitored |
| **c. Monitors the environment for safety and availability of resources (e.g., equipment)** | **Item 4c – removed**  **Comment:**  **This item was removed because it is not applicable to in-hospital VR scenarios conducted in a safe environment with a predefined and limited set of available equipment and resources.** |
| **d. Monitors progress toward the goal and identifies changes that could alter the plan of care** | **4.3 Discusses treatment progress based on the patient’s condition.**  5 = Treatment goals are clearly defined based on patient condition (e.g., using ABCDE) with necessary actions  3 = Patient condition guides a rough direction for treatment  1 = No treatment goals are defined |
| **e. Fosters communication to ensure that team members have a shared mental model** | **Item 4e – removed**  **Comment:**  **The establishment of a shared mental model is sufficiently captured through the assessment of team huddles (adapted Item 3.2) and verbalization of treatment progress (adapted Item 4.2).** |
| **5. Mutual Support** |  |
| **a. Provides task-related support and assistance** | **5.1 Provides task-related support and assistance when needed.**  5 = Support is consistently offered in appropriate situations and matches the team member’s competence  3 = Support is occasionally or insufficiently offered  1 = No support is offered |
| **b. Provides timely and constructive feedback to team members** | **5.2 Provides timely and constructive feedback to team members.**  5 = Feedback consistently meets good-feedback criteria (timely, constructive, action-oriented)  3 = Feedback only partially meets good-feedback criteria  1 = Feedback is absent or inappropriate, even when warranted |
| **c. Effectively advocates for patient safety using the Assertive Statement, Two-Challenge Rule,or CUS** | **5.3 Alerts to patient deterioration and raises concerns a second time if ignored (Two-Challenge Rule).**  5 = Two-Challenge Rule is applied appropriately  3 = Concerns are vague or only raised once despite lack of acknowledgement  1 = Concerns are not raised despite the situation would require it. |
| **d. Uses the Two-Challenge Rule or DESC Script to resolve conflict** | **Item 5d – removed**  **Comment:**  **The DESC script represents a form of structured conflict communication derived from nonviolent communication principles. It is comparatively complex, more difficult to teach in short team training sessions, and considerably less relevant in dyadic teams with clearly defined roles. The Two-Challenge Rule is already represented by adapted Item 5.3; therefore, the original Item 5d was removed.** |
