# Supplementary material for: Adapting the TeamSTEPPS team performance observation tool for dyadic interprofessional VR simulations (vTPOT): a multi-step validation study
Source: Adv Simul (Lond). 2026 Mar 12;11:24. doi: 10.1186/s41077-026-00431-0 (PMC13023181; doi:10.1186/s41077-026-00431-0)

## vTPOT Rater Codebook for the VISTA Study

**General Note:**
The following consensus serves as a supplement to the existing vTPOT item texts in the context of the VISTA study, for rating emergency scenarios in STEP-VR in virtual reality. Intermediate descriptors (“2”) are provided for rater orientation only and do not constitute additional scoring levels.

**2.1 Codebook Consensus**

All relevant clinical information (e.g., vital signs, lab results, physician orders) is rated equally, regardless of whether the team member is physically present or not.
Information is considered timely if communicated within one minute. If team members are in different rooms, the one-minute window starts once they are in the same room again.

**2.2 Codebook Consensus**

A “standard list” serves as a guideline, which can be extended with additional scenario-relevant sources as needed (e.g., urine status/culture in the UTI scenario, thermometer readings in the infection case).

- **5 points**: “Complete source check” — history, physical examination, monitor, nursing/initial notes, lab, imaging (+ any additional scenario-relevant sources).
- **3 points**: Only “obvious sources” are considered (e.g., history, vital signs monitor, lab).

Sources should be actively considered, not just observed. Correct medical interpretation is **not** required for this item.

**2.3 Codebook Consensus**

Checkbacks should focus only on high-risk situations (medications, infusions, oxygen administration including ventilation). General information is not considered. Circulatory data or lab values are also not included.
If no high-risk information is communicated at all, mark with X.

**2.4 Codebook Consensus**

In practice, for VISTA scenarios, rate with X as handovers are generally not required.

**3.2 Codebook Consensus**

Only **huddles** are considered for this item.

- **5**: Ideally, an initiating sentence like “Let’s discuss” and appropriately synthesizes findings, patient condition, and next steps.
- **3**: Intention is generally clear, but not explicitly stated or fully implemented.
- **2**: Intention is clear but team members are not adequately included.

Positive credit if a synthesis of information and outlook occurs, resembling a debriefing. (1UP)

**3.3 Codebook Consensus**

Rate the **leading (physician) person**.

- **5 — Integration**: Suggestions are acknowledged and addressed in a professional, action-oriented manner.
- **3 — Active listening**: Verbal acknowledgment **and** minimal reference to content.
- **2 — Sporadic or monosyllabic response**.

**4.1 Codebook Consensus**

Regular monitoring may include scenario-specific assessments of pain, fever, blood pressure/heart rate, O₂ saturation, and optionally hemoglobin.
Merely observing a monitor without verbalization does **not** count as sufficient re-evaluation.

**4.2 Codebook Consensus**

Focus more on verbal communication:

- Ask whether relevant actions were carried out.
- Confirm what the team member is currently doing and whether the agreed plan is followed.
- Late detection of omissions should be rated negatively.

**4.3 Codebook Consensus**

Distinct from huddles (summary and overarching), here we continuously define goals and actions.

- **5**: Maximum clarity with concrete numbers.
- **3**: Reasonably clear direction with general categories.
- **1**: Missing, purely reactive, or obviously unplanned approach.

**Scenario-specific behavioral anchors (simplified version):**

- **5**: Clear, patient-centered treatment goals are formulated, temporally or quantitatively specified, directly derivable from patient condition. (Example: “MAP should reach ≥65 mmHg within 10 min; therefore, give 500 ml Jonosteril.”)
- **3**: Broad direction or priority given; goals not precisely timed or quantified. (Example: “We should stabilize circulation; give vasopressors.”)
- **1**: No discernible treatment goal; actions appear reactive or unplanned. (Example: “Oh, BP is low… do something.”)

**5.1 Codebook Consensus**

Technical actions (e.g., VR usability issues) are also considered; the outcome does not affect rating.
Explicit requests for help or explicit offers are quality criteria.
Collaborative problem-solving is **not** penalized.
Support **not offered despite clear need** is rated negatively.

**5.2 Codebook Consensus**

The end-of-scenario debrief is included as a feedback-worthy situation (feedback is always appropriate).

- Lack of feedback during debrief is **not** rated with X.
- Evaluate person-specific behavioral feedback (“I liked that you did XYZ”) according to the stated criteria if it occurs.
- Merely mentioning completed or uncompleted goals is **insufficient**.
- Feedback from the end-debrief, if it occurred, is approximately credited 1:1 with prior scenario feedback-worthy situations.
- Missing feedback during the scenario is only rated for omitted or incorrect actions in critical situations (e.g., essential medication/O₂/blood transfusion/infusion omitted).

**5.3 Codebook Consensus**

Each scenario includes at least one “challenge-worthy” situation (e.g., dropping BP or SpO₂). Follow the flowchart for rating.
The Two-Challenge Rule applies in cases of serious medication errors (e.g., contraindicated medication like heparin in bleeding, aminopenicillin in allergy cases).


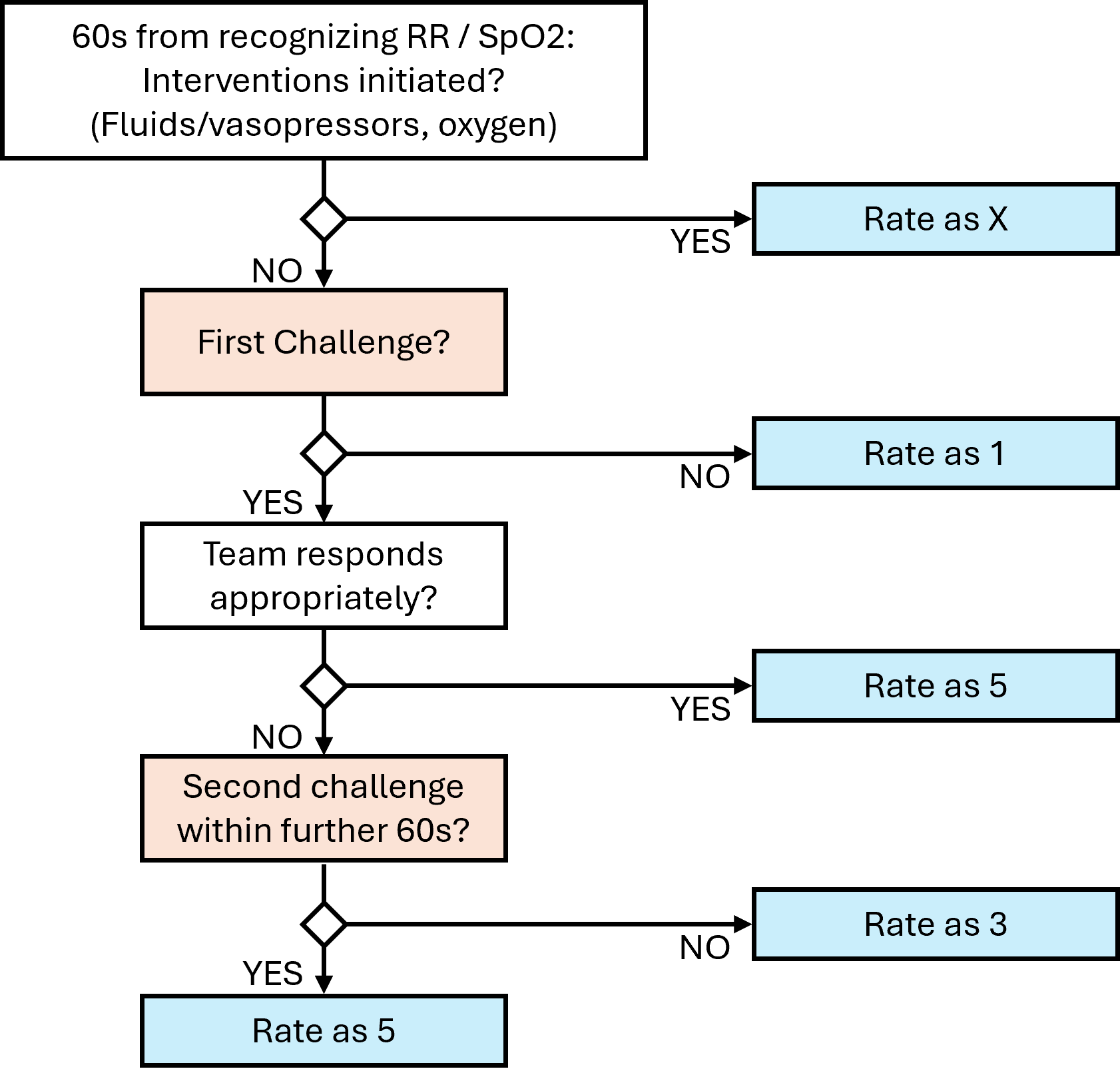

Supplement: Supplementary file 4 — Supplementary Material 4. Table S4: Comparison of TPOT and vTPOT Items. [file 41077_2026_431_MOESM4_ESM.docx]
